# Supplementary material for: Age and Sex as Determinants of Acute Domoic Acid Toxicity in a Mouse Model
Source: Toxins (Basel). 2023 Apr 1;15(4):259. doi: 10.3390/toxins15040259 (PMC10143184; doi:10.3390/toxins15040259)
Supplement: Supplementary file 1 [file toxins-15-00259-s001.zip › toxins-2223287-supplementary.pdf]

Supplementary Materials

Age and Sex as Determinants of Acute Domoic Acid Toxicity in a Mouse Model

Table S1. Estimated hazard ratios (HRs) and *p*-values associated with variables included in each Cox proportional hazard (CPH) regression model for clonic-tonic convulsions * $p < 0.05$.

Models and model variables	Concordance	Estimated HRs	*p*-values
Additive	91%		
Dose		10.0 [1.9, 52]	0.0062 *
Sex		0.29 [0.31, 2.75]	0.28
Single-variable dose	90%		
Dose		16.3 [2.9, 90.4]	0.0015
Single-variable sex	72%		
Sex		0.12 [0.015, 1.0]	0.050

Table S2. Likelihood ratio test (LRT) *p*-values comparing nested single-variable CPH model fit to additive multivariable CPH model fit for clonic-tonic convulsions *** $p < 0.0005$.

Full model	Nested model	LRT *p*-values comparing full model to nested model
Dose + Sex	Dose	0.24
Dose + Sex	Sex	0.00020 ***

Table S3. Estimated HRs and *p*-values associated with variables included in each CPH regression model for hindlimb tremors * p <0.05, ** p <0.005, *** p <0.0005.

Models and model variables	Concordance	Estimated HRs	*p*-values
Interaction			
Dose * age + sex	84.5%		
Dose		10.0 [2.0, 50.0]	0.0053 *
Age		36.0 [0.84, 1540.1]	0.616
Sex		0.31 [0.11, 0.87]	0.026 *
Dose:age		0.29 [0.051, 1.65]	0.16
Dose * sex + age	84.9%		
Dose		3.6 [1.81, 7.0]	0.00024 ***
Age		3.1 [1.3, 7.2]	0.011 *
Sex		0.18 [0.0081, 3.81]	0.27
Dose:sex		1.37 [0.23, 8.065]	0.73
Sex * age + dose	85.1%		
Dose		3.72 [2.00, 7.00]	$3.9e^{-5}$ ***
Age		2.83 [1.10, 7.25]	0.030 *
Sex		0.21 [0.025, 1.71]	0.14
Sex:age		1.61 [0.15, 17.44]	0.70
Additive			
Dose + age + sex	85.3%		
Dose		3.74 [2.00, 6.99]	$3.6e^{-5}$ ***
Age		3.07 [1.30, 7.24]	0.010 *
Sex		0.29 [0.11, 0.81]	0.019 *
Dose + age	83.8%		
Dose		1.57 [2.47, 9.26]	$3.4e^{-6}$ ***
Age		2.85 [1.21, 6.68]	0.016 *
Dose + sex	82.1%		
Dose		1.23 [1.83, 6.45]	0.00012 ***
Sex		0.32 [0.11, 0.88]	0.028 *
Sex + age	72.0%		
Sex		0.20 [0.75, 0.54]	0.0015 **
Age		2.41 [1.03, 5.64]	0.043 *
Single-variable			
Dose	79.1%		
Dose		4.43 [2.30, 8.53]	$8.6e^{-6}$ ***
Age	60.1%		
Age		2.29 [0.98, 5.36]	0.055
Sex	67.1%		
Sex		0.21 [0.078, 0.56]	0.0018 **

Table S4. Likelihood ratio test *p*-values comparing nested single-variable CPH model fit to additive multivariable CPH model fit for hindlimb tremors * p <0.05, ** p <0.005, *** p <0.0005.

**Full model**	**Nested model**	**LRT *p*-values comparing full model to nested model**
Dose + Age	Dose	0.013 *
Dose + Sex	Dose	0.018 *
Age + Dose	Age	$1.3e^{-7}$ ***
Age + Sex	Age	0.00036 ***
Sex + Dose	Sex	$1.4e^{-5}$ ***
Sex + Age	Sex	0.036 *
Dose + Age + Sex	Dose + Sex	0.0078 **
Dose + Age + Sex	Dose + Age	0.011 *
Dose + Age + Sex	Age + Sex	$3.4e^{-6}$ ***

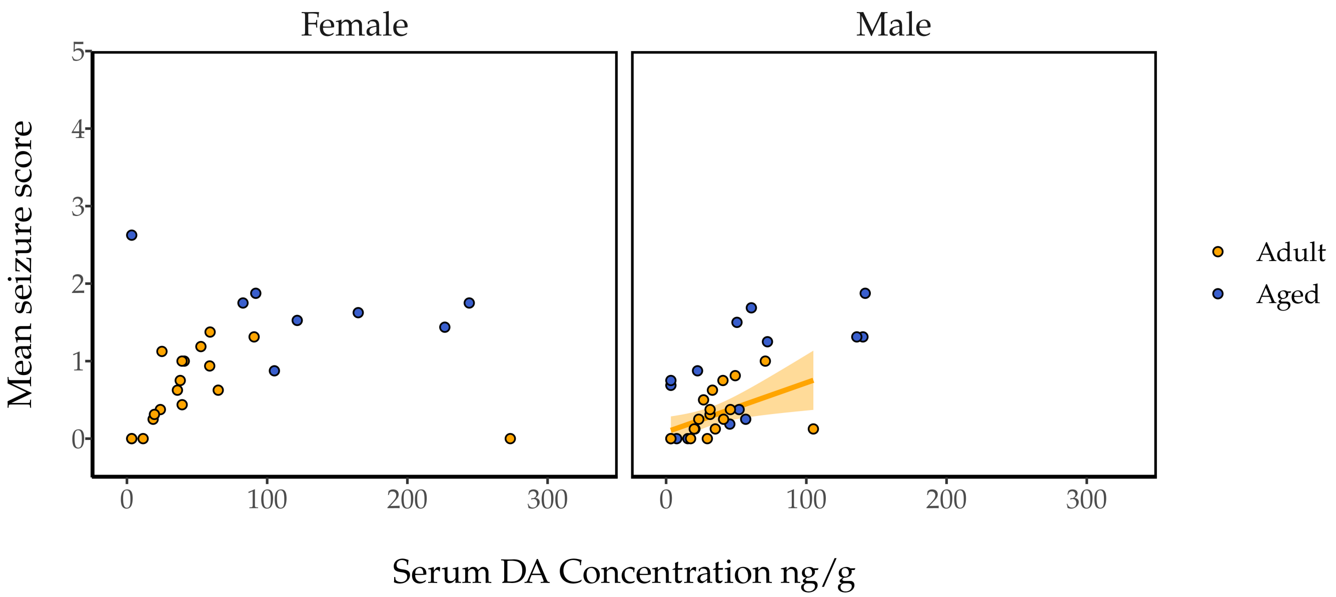

Figure S1. Regression analyses of mean seizure score versus serum domoic acid (DA) concentrations indicated that serum DA level was positively associated with mean seizure score in adult male mice only. Overall linear regression model for adult male mice was statistically significant ($F(1, 18) = 6.51$, $p = 0.020$). For every 100 ng/g greater serum DA concentration, mean seizure score increased by 0.64 (± 0.25; $p = 0.020$).
